# Deep structure, long‐distance migration and admixture in the colour polymorphic land snail *Cepaea nemoralis*

**DOI:** 10.1111/jeb.14060

**Published:** 2022-07-13

**Authors:** Daniel Ramos‐Gonzalez, Suzanne V. Saenko, Angus Davison

**Affiliations:** ^1^ School of Life Sciences University of Nottingham Nottingham UK; ^2^ Evolutionary Ecology Naturalis Biodiversity Center Leiden The Netherlands; ^3^ Institute of Biology Leiden Leiden University Leiden The Netherlands

**Keywords:** *Cepaea*, colour, polymorphism, snail

## Abstract

Although snails of the genus *Cepaea* have historically been important in studying colour polymorphism, an ongoing issue is that there is a lack of knowledge of the underlying genetics of the polymorphism, as well as an absence of genomic data to put findings in context. We, therefore, used phylogenomic methods to begin to investigate the post‐glacial history of *Cepaea nemoralis*, with a long‐term aim to understand the roles that selection and drift have in determining both European‐wide and local patterns of colour polymorphism. By combining prior and new mitochondrial DNA data from over 1500 individuals with ddRAD genomic data from representative individuals across Europe, we show that patterns of differentiation are primarily due to multiple deeply diverged populations of snails. Minimally, there is a widespread Central European population and additional diverged groups in Northern Spain, the Pyrenees, as well as likely Italy and South Eastern Europe. The genomic analysis showed that the present‐day snails in Ireland and possibly some other locations are likely descendants of admixture between snails from the Pyrenees and the Central European group, an observation that is consistent with prior inferences from mitochondrial DNA alone. The interpretation is that *C. nemoralis* may have arrived in Ireland via long‐distance migration from the Pyrenean region, subsequently admixing with arrivals from elsewhere. This work, therefore, provides a baseline expectation for future studies on the genetics of the colour polymorphism, as well as providing a comparator for similar species.

## Graphical abstract

Population genomics of the colour polymorphic snail *Cepaea neamoralis*.

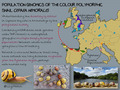

## 1 INTRODUCTION

The study of animal colour has been a key in providing evidence for some of the central tenets of biology, especially with respect to genetics and evolution. For example, early work on the inheritance of colour traits contributed to an understanding of Mendelian genetics (Staples‐Browne, 1908; Wheldale, 1907). Subsequent studies on the distribution and predation of colour morphs shaped our understanding of how natural selection may operate in wild populations, with perhaps some of the most important insights coming from moths and snails (Cook, 2003; Jones et al., 1977). In the 21st century, DNA sequencing technologies have been used to identify the underlying genes that determine variation in colour morphs within species and their evolutionary history (Wellenreuther et al., 2014). These same methods have also enabled the subdiscipline of phylogeography to move away from the use of one or a few markers to studies that involve thousands of genetic markers (Dussex et al., 2020; Kotlik et al., 2018; Lucena‐Perez et al., 2020). Such studies have revealed how connectivity between populations, or lack of, shapes, the genetic structure of species, and critically, how regions of the genome respond differently depending upon the nature of selection and the genetic architecture of a particular colour trait (e.g. Pardo‐Diaz et al., 2012; Poelstra et al., 2014).

**FIGURE 1:**
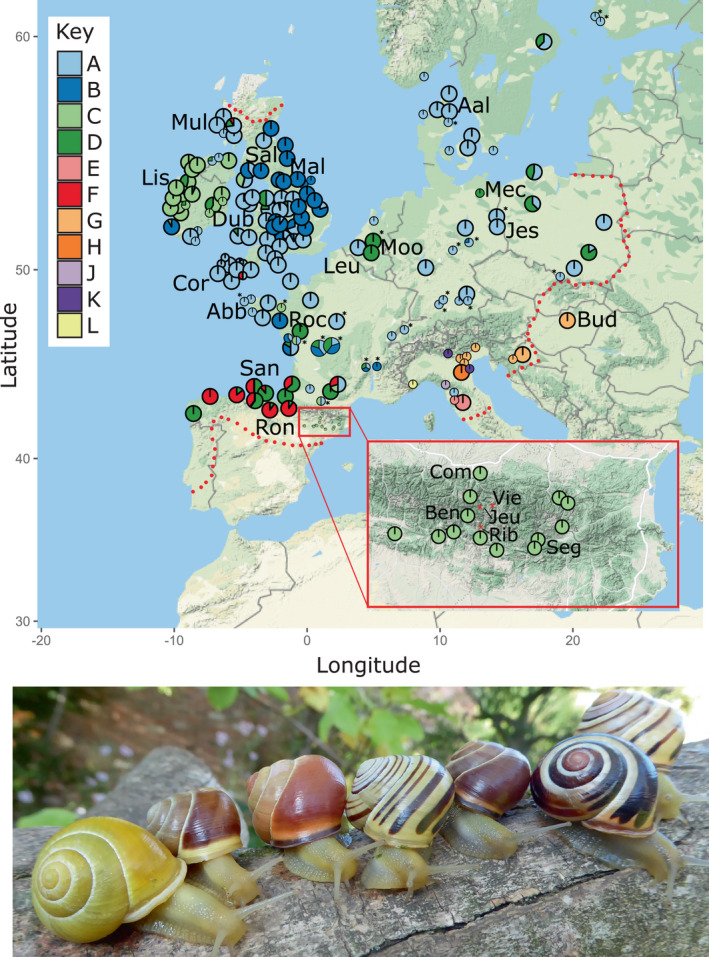
Top: European map summarizing the results of the mtDNA study on *C. nemoralis*, as well as the locations and abbreviation code for the samples used for the ddRAD study. Pies are coloured according to mtDNA lineage (see Figure S1); larger pies indicate *n* > 5 per site. Limits of the present‐day distribution of *C. nemoralis* shown by the red dotted line. Data from Layton et al. (2019) indicated by asterisk. Bottom: Some of the *C. nemoralis* colour and banding morphs, from left: (1) yellow unbanded, white lip; (2) brown mid‐banded, white lip; (3) pink unbanded, normal lip; (4) yellow five‐banded, white lip; (5) brown unbanded, normal lip; (6) pink five‐banded, normal lip; (7) yellow three‐banded (10 305), normal lip. Image credit: Angus Davison

Historically, some of the most important animals in studying colour polymorphism have been the two snail species, *Cepaea nemoralis* and *C. hortensis*. One benefit of working with these species is that variation in colour and banding (Figure 1) shows straightforward inheritance (Cook, 2017; Jones et al., 1977; Richards et al., 2013), being controlled by a series of at least nine loci, of which five make a single ‘supergene’ containing tightly linked colour, banding and other loci. In these two species, we now have some understanding of the pigments and shell proteome (Affenzeller et al., 2020; Mann & Jackson, 2014; Williams, 2017) and some knowledge underlying the development of the banding pattern (Jackson et al., 2021) and have begun to use genomic methods to try to map and identify the genes involved (Kerkvliet et al., 2017; Richards et al., 2013).

However, although ongoing studies on snails in general continue to provide evidence for the relative role of various forms of natural selection and random drift in promoting and maintaining variation, snails (and molluscs in general) are poorly represented in phylogeographic or population genomic studies. For example, most studies on terrestrial snails have been limited to using just a few genes, even those published recently (de Weerd et al., 2016; Ebbs et al., 2018; Neiber et al., 2017). In our own work on *C. nemoralis*, we sampled 880 individuals from 111 locations, but used only one genetic marker, a fragment of the mitochondrial gene cytochrome c oxidase subunit 1 (Grindon & Davison, 2013). Otherwise, there are just a few notable exceptions (Haponski et al., 2019; Hirano et al., 2019; Miura et al., 2020; Neiber & Hausdorf, 2015b; Westram et al., 2018), with a few studies in Europe (Koch et al., 2017; Razkin et al., 2016; Xu & Hausdorf, 2021), and a single genome re‐sequencing study (Chueca et al., 2021a). This is a problem because understanding colour polymorphism requires a contextual understanding of the history of populations in general, and more precisely, a gene‐by‐gene account of the impact that drift and selection have had upon the genome.

One issue for *Cepaea* snails is that Jones et al. (1977) questioned whether understanding the polymorphism is ‘a problem with too many solutions?’, which led to difficulties arguing the case for these snails as a study organism, or molluscan ‘model’ (Davison & Neiman, 2021). Actually, the misunderstood intention of Jones et al. (1977) was to emphasize the perfect case study provided by *Cepaea*. Simple explanations for phenotypic variation, including colour, are likely an exception, so they were making the point that it is important to study organisms for which polymorphism may be explained by a variety of processes, precisely because they are more representative. Given that present‐day genomic technologies should allow us to uncover the relative contributions of each of these processes in making contemporary diversity, this aim should now be realizable. Unfortunately, a second general issue is that extracting DNA from snails is sometimes problematic (Adema, 2021), and snails in general have large and repetitive genomes, which are difficult to assemble. Advances in the understanding of the colour polymorphisms of snails have, therefore, tended to lag behind other species, especially invertebrates with relatively small genomes (Davison & Neiman, 2021).

In this study, we take advantage of a first draft genome sequence of *C. nemoralis* (∼3.5 Gb, 28 537 contigs with N50 of 333 kb; Saenko et al., 2021) and restriction site‐associated DNA sequencing methods (ddRAD) to understand the phylogeographic and population genomic structure of *C. nemoralis*. By combining the genomic data with mtDNA sequences generated from three prior studies (Davison, 2000; Grindon & Davison, 2013; Layton et al., 2019) and new sequences from Italy, the proximate aim is to begin to understand the post‐glacial history of the species. The ultimate aim is to provide context for subsequent studies on the evolutionary origins of the supergene and the relative roles that natural selection and drift may play in the establishment and loss of colour polymorphism in local populations.

## 2 MATERIALS AND METHODS

### 2.1 Sample collection

In previous work, we and others collected *C. nemoralis* from across Europe, euthanizing snails by freezing at −80°C upon arrival at the University of Nottingham (Davison, 2000; Grindon & Davison, 2013). In this study, we strategically sampled from this prior collection, including individuals from Germany, Belgium, France, Hungary, Denmark, Spain, UK and Ireland. We also used snails from a 2017/2018 collection (Ramos Gonzalez & Davison, 2021) from three valleys in the Pyrenees, Valle de Vielha, Valle de Jueu and Valle Noguera Ribagorzana (subsequently “Vie”, “Jue” and “Rib”).

For the mitochondrial DNA analysis, we combined the data from our own prior studies (Davison, 2000, Grindon & Davison, 2013) with data from another recently published work (Layton et al., 2019), the latter of particular benefit because the study had good coverage of sites in central France. In addition, we also added new data from samples from Italy, useful because the peninsula was a potential refugium during previous glaciations but poorly sampled in the original studies.

For the genomic analyses, individuals were selected to ensure a wide geographic spread and to include representatives of different colour morphs, as well as the major mitochondrial lineages reported in the prior studies (Figure 1, Table 1).

**TABLE 1:** Summary showing the locations and phenotypes of the individual samples used for the analyses of genomic variation, including mitochondrial haplotype (Grindon & Davison, 2013); “group” reflects the clustering returned using Gaussian finite mixture modelling

Location			ID	Grindon ID	Collector	Date	Latitude	Longitude	mtDNA	Group	Colour	Banding
Belgium	Leuven	Leu	40	Kesscn5	Thierry Backeljau	6/29/2005	50.883467	4.722461	D39	8	Pink	00300
			45	Kesscn8		6/29/2005			D8	8	Yellow	00000
	Moortsele	Moo	48	Ooscn2a	Thierry Backeljau	7/27/2005	50.958811	3.779839	A27	10	Yellow	12 345
Denmark	Aalborg	Aal	46	Aal2cn5	Adele Grindon	7/20/2006	57.051833	9.872750	A51	10	Yellow	12 345
			47	Aal2cn7		7/20/2006			A22	10	Pink	12 345
England	Cornwall	Cor	26	Coascn3	Elizabeth Bailes	6/18/2005	50.254919	−5.043106	A87	10	Yellow	00000
			66	Coascn5		6/18/2005			A87	10	Yellow	00000
	Malham	Mal	53	Malcn3	Adele Grindon	8/10/2005	54.119917	−2.180333	B8	10	Yellow	12 345
			54	Malcn5		8/10/2005			B8	10	Yellow	00000
	St. Bees	Sal	52	Saltcn9	AD	5/2/2007	54.489256	−3.589572	B3	10	Pink	00300
France	Comminges	Com	63	Romancn5	Adele Grindon	4/24/2007	43.027983	0.722267	C59	5	Yellow	00345
			64	Romancn7		4/24/2007			C56	5	Yellow	00000
	La Roche	Roc	31	Berncn2	Adele Grindon	4/23/2007	47.511767	−2.285667	B23	7	Pink	12 345
	Pont L'Abbe	Abb	28	Abbecn1	Adele Grindon	4/24/2007	47.883950	−4.182567	A58	10	Yellow	00300
			29	Abbecn3		4/24/2007			A85	10	Yellow	00300
Germany	Jessern	Jes	49	Jesscn1	Malgorzata Ozgo	5/23/2005	52.016667	14.183333	A11	10	Pink	00000
			51	Jesscn9		5/23/2005			A40	10	Pink	00300
	Mecklenburg	Mec	50	Robcn2	Michael Zettler	5/29/2005	53.581667	12.985000	D33	4	Pink	00300
Hungary	Budapest	Bud	42	Hungcn4a	Feher Zoltan	9/13/2005	47.425808	19.448261	G11	1	Yellow	12 345
			43	Hungcn5a		9/13/2005			G13	1	Yellow	00300
			44	Hungcn1a		9/13/2005			G12	1	Yellow	00300
Ireland	Dublin	Dub	38	Dublincn4	AD	5/3/2006	53.318475	−6.251375	C15	7	Pink	12 345
			39	Dublincn2		5/3/2006			C15	7	Yellow	00345
	Lisdoonvarna	Lis	36	Liscn1	AD	4/7/2007	53.026317	−9.290167	B27	7	Yellow	12 345
			37	Liscn2		4/7/2007			C8	7	Pink	12 345
Scotland	Mull	Mul	27	Mullcn4	Max Cooper	6/13/2007	56.578339	−6.283422	A47	10	Pink	12 345
Spain	Benasque	Ben	61	Bencn3	Adele Grindon	4/16/2007	42.599367	0.527000	C19	9	Yellow	12 345
			62	Bencn1		4/16/2007			C17	9	Yellow	00000
	Jueu	Jue	34	DRG106_15	DRG	6/29/2018	42.677900	0.705500	‐	9	Yellow	00000
			56	DRG103_5		6/29/2018			‐	9	Pink	00000
	Ribagorzana	Rib	57	DRG77_14b	DRG	10/12/2017	42.479200	0.714000	‐		Yellow	00000
			58	DRG124_16		6/29/2018			‐		Pink	00000
	Roncesvalles	Ron	41	Ron2cn3	Adele Grindon	4/14/2007	43.009300	−1.319067	F41	3	Pink	12 345
	Santander	San	60	Santacn8	AD	10/21/2006	43.414400	−3.741300	D52	2	Yellow	00000
			65	Santacn9		10/21/2006			F25	2	Yellow	00000
			30	Playacn9	Adele Grindon	4/12/2007	43.487067	−3.792683	D49	2	Yellow	00000
			35	Playacn10		4/12/2007			D47	2	Yellow	00000
			55	Playacn7		4/12/2007			F24	2	Yellow	00000
	Segre	Seg	59	Serge2cn1a	Adele Grindon	5/23/2006	42.359817	1.479550	C1	6	Yellow	12 345
	Vielha	Vie	32	DRG17_9	DRG	10/12/2017	42.690500	0.874200	‐	9	Yellow	12 345
			33	DRG17_12		10/12/2017			‐	9	Pink	00000

### 2.2 DNA methods

High‐molecular‐weight genomic DNA was extracted from frozen snail foot tissue, using methods similar to those described previously (Gonzalez et al., 2019; Richards et al., 2013). In brief, tissue slices were incubated at 65°C in lysis buffer (10 mM Tris, 0.1 M EDTA, 0.5% SDS), with proteinase K (0.2 mg/ml), with occasional inversion. RNase A (80 μg/ml) was added, and then, each tube was incubated for one more hour. The mixture was cooled to room temperature and then extracted using the standard phenol/chloroform/iso‐amyl ethanol (25:24:1) method, with ethanol precipitation. DNA quality was visually verified by agarose gel electrophoresis and then quantified and further checked using a NanoDrop 2000c spectrophotometer and a Qubit 2.0 Fluorometer. ddRAD libraries were generated by SNPsaurus (https://www.snpsaurus.com/), using the method of Russello et al. (2015) and 75 ng of input DNA. Using a service provided by SNPsaurus at the University of Oregon (https://www.snpsaurus.com/), one lane of a HiSeq4000 was then used to generate 150 base pair sequence reads, which were then demultiplexed and stored as compressed fastq files. In addition, a ∼600 base pair (b.p.) fragment of the mitochondrial gene COI was amplified by the polymerase chain reaction (PCR) for some individuals, using either the Folmer et al. (1994) primers, those of Gittenberger et al. (2004) or a combination of the two, as described previously (Grindon & Davison, 2013). The PCR product was then sequenced with forward primers (and reverse, if necessary) using BigDye™ Terminator version 3.0 Cycle Sequencing Kit.

### 2.3 Analyses of mitochondrial DNA variation

The randomized axelerated maximum likelihood method, as implemented in raxml 8.2.12 (Stamatakis, 2014), was used with a partitioned data set (one for the 1st/2nd codon positions and another for the 3rd codon position) and produced a mitochondrial phylogeny, with rapid bootstrap resampling (100 replicates). The phylogeny was then used to place individual haplotypes into lineages, as previously described (Grindon & Davison, 2013).

### 2.4 Quality checking, variant calling and filtering

fastqc v0.11.9 software was used to check the quality of the reads, and then, adapters were removed using trimmomatic 0.39 (Bolger et al., 2014). The fastq files were then aligned to the draft genome (Saenko et al., 2021) using the Burrows‐Wheeler Alignment method (Li et al., 2009) in bwa v0.7.17‐r1188, using default settings and marking low‐quality alignments (−M). samtools v1.11 was then used to sort and compress the sam files to bam files, then bcftools v1.10 (Danecek & McCarthy, 2017) was used to call variants. vcftools (Danecek et al., 2011) was initially used to explore the unfiltered data, to guide subsequent filtering thresholds (e.g. see https://speciationgenomics.github.io/filtering_vcfs/ or the Davison github repository). A filtered data set of bi‐allelic loci was then created, retaining those sites where there was a missing call in <25% of individuals, a mean sequencing depth across all individuals of between 0.25 and 25 reads (removes false positives and avoids repetitive regions), a minimum quality score of 20 and minor allele frequency (MAF) of 0.1, the latter based on an inspection of the distribution of allele frequencies. PCR duplication was checked using a script ‘checkHetsIndvVCF.sh’, originally written by David Marques and edited by Joana Meier (https://github.com/alphaneer/scripts‐1/blob/master/checkHetsIndvVCF.sh; Meier et al., 2018).

### 2.5 Analyses of genomic variation

We used principal component analysis (Patterson et al., 2006), whole‐genome phylogeny and admixture tests to analyse the relationships between the individuals.

First, as others have done (Bryc et al., 2010; Decker et al., 2014; Marques et al., 2022), principal components analysis (PCA) and Gaussian finite mixture modelling methods were used as an independent means of exploring the broad phylogenomic relationship between individuals, independent of models of sequence evolution. For the PCA, but not other analyses, the filtered variant calling file was first pruned using plink v1.9 (Purcell et al., 2007), then linkage‐pruned sites were used to generate eigenvalues and vectors. The PCA output was plotted in r version 3.4.1 (R Core Team, 2020) using tidyverse v1.3.0 (Wickham et al., 2019). To identify possible clusters within the data, Gaussian finite mixture modelling was applied to the PCA values using mclust 5.4.6 (Scrucca et al., 2016), assuming between 1 and 20 possible clusters. The best‐fitting model was then determined using the Bayesian Information Criteria (BIC), with significant differences established by using a bootstrap likelihood ratio test, assuming varying orientations and homogeneity of variance.

To understand the distribution of genomic variation by geography, pairwise values of Weir & Cockerham's *F*_st_ between populations were estimated using vcftools (Danecek et al., 2011). To achieve this, genome‐wide *F*_st_ was used directly by plotting against geographic distance or transformed into a dissimilarity matrix, using the vegan 2.5‐6 package in r (Jari Oksanen et al., 2018) and again plotted against geographic distance. Significance was tested using a Mantel test based on Spearman's rank correlation and 9999 permutations, again using vegan 2.5‐6.

In the future—when whole‐genome analyses are possible and the colour and banding loci have been finely mapped—we aim to understand how gene flow impacts upon different regions of the genome, for example, using a window‐based approach. As a starting point, we estimated *F*_st_ by contig and also estimated Mantel's *r* statistic for the *F*_st_ for each contig and all pairwise comparisons. To achieve this, the same analyses as above were carried out on each of the individual contigs, again testing significance using a Mantel test.

To further understand the phylogenomic relationship between individuals, the concatenated biallelic SNP ddRAD data were used to generate a phylogeny, using raxml v8.2.9 with the GTRGAMMA model and 100 bootstrap replicates (Stamatakis, 2014).

The program admixture v1.3.0 was used to determine the best number of clusters (*K*), by computing the fivefold cross‐validation error (Alexander & Lange, 2011), with the aim of then using treemix 1.13 (Pickrell & Pritchard, 2012) to investigate possible migration events.

To support these analyses, *D* statistics were used to explore evidence of introgression between Pyrenean and central European snails, using admixtools version 6.0 (Patterson et al., 2012) and admixr version 0.7.1 (Petr et al., 2019). Leave‐one‐out cross‐validation was used to determine the best‐supported value, with significance evaluated using block jackknife and the *Z*‐score.

For the *D* statistics, the underlying principle (Ravinet & Meier, 2020) is that if W, X, Y, Z represent four taxa with a relationship (([W,X,]Y),Z), with no hybridization, then the number of discordant SNPs grouping W and Y together (BABA patterns) is expected to be roughly the same as the number of SNPs grouping X and Y together (ABBA). Therefore, we set out to test whether the Irish samples and others show evidence of introgression from focal populations; groupings and analyses were based on prior PCA, admixture analyses and RAxML phylogenies. Using Hungarian samples as an outgroup (Z), we compared individual central European populations (W; England: Cornwall, St. Bees, Malham; Germany: Jessern; Belgium: Moortsele; Denmark: Aalborg) and individual central Pyrenean populations (Y; Vielha, Jueu, Benasque, Segre and Comminges) to the populations of interest (X; Ireland: Lisdoonvarna, Dublin; France: La Roche; Scotland: Mull).

## 3 RESULTS

### 3.1 Mitochondrial DNA

Here, we combined data from two prior studies (Davison, 2000; Grindon & Davison, 2013) with a new study (Layton et al., 2019). We also added 17 individuals from a further nine locations in Italy, making a combined total of 1521 individuals from 180 locations across Europe (Table S1). The revised mtDNA phylogeny (Figure S1; rooted using the sister taxon *C. hortensis*) shows the same pattern as before (Figure 1), with the new finding that Italy has several lineages that are not found anywhere else, except in Croatia and Hungary. The majority of Europe (Figure 1) has snails with mtDNA lineages A, B, C, D and F, with the other lineages relatively rare and/or range restricted.

### 3.2 Restriction site‐associated DNA sequencing

ddRAD libraries were prepared from 41 snails and 22 locations across Europe (Table 1). The locations used commonly have mtDNA lineages A (Aal, Abb, Cor, Jes, Leu, Mul), B (Lis, Mal, Roc, Sal), C (Ben, Com, Dub, Jeu, Lis, Rib, Seg, Vie), D (Dub, Mec, Moo, Mul, Ron, San), F (Ron, Sand), G (Bud).

After discarding two samples due to a low number of reads (S57 and S58 both from Ribagorzana valley in Spain; Table S2), 39 individuals were put through to the main analysis. An average of 95% of reads mapped to the reference genome (Table S2). After filtering, the whole‐genome data set was reduced to 8689 bi‐allelic loci contained on 2323 genome contigs, making a total length of about 990 Mb (out of 28 538 contigs in the reference genome of ~3.5 Gb; Table S3). There was a mean of 3.74 SNPs per contig and, as expected, a correlation between contig length and number of SNPs (*r* = 0.11, *p* < 0.001; Figure S2). There was no evidence for PCR duplication problems.

### 3.3 Different genomic evolutionary history of the colour and geographic variation

**FIGURE 2:**
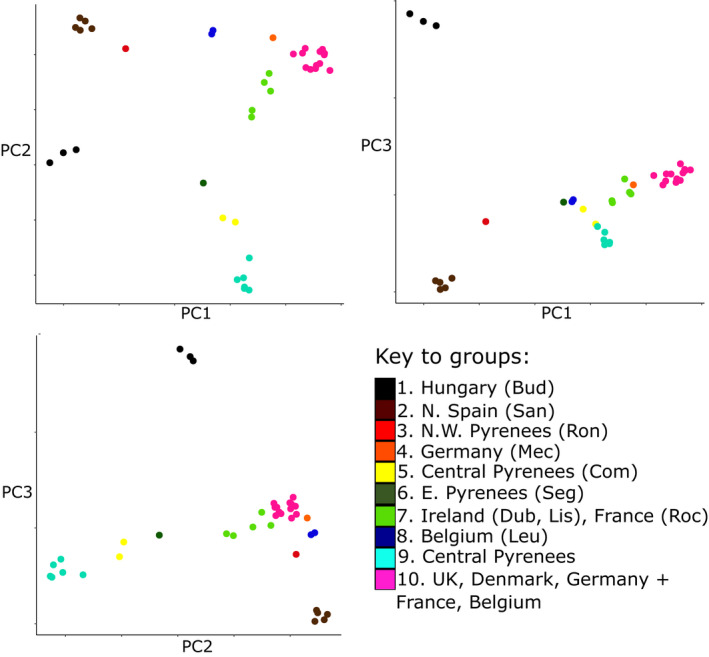
First three axes of principal component analysis of the whole‐genome data of individual *Cepaea nemoralis* snails using 8689 biallelic loci. Individuals are coloured according to the clusters defined by Gaussian finite mixture modelling. Groups 1 to 6 and 8 are made up of snails from single sample sites; group 7 samples from Ireland (Lis, dub) and NW France (roc); group 9 samples from the Central Pyrenees (ben, Jue, vie, rib), with group 10 all remaining samples (UK: Mul, Sal, mal, Cor; France: Abb; Belgium: Moo, Denmark: Aal; Germany: Jes)

A principal components analysis of the whole‐genome data showed that individuals of *C. nemoralis* broadly cluster according to geography (Figure 2) with PC1, PC2 and PC3 together explaining 37% of the variance (Table S4). Samples from Hungary (Budapest, *n* = 3), North‐West Spain (Santander, *n* = 5) and the West Pyrenees (Roncesvalles, *n* = 1) were separated from the other samples on PC1 (Figure 2a; PC1 = 15%). PC3 separated the Hungarian samples from the North‐West Iberian samples, with the others intermediate (Figure 2b; PC3 = 10%). PC2 separated the populations found in the Central Pyrenees (Vielha, Jueu & Benasque, n = 6; Comminges, n = 2; Segre, n = 1) from samples found elsewhere (Figure 2c; PC2 = 12%). Of the other samples, those from Budapest (*n* = 3) and from Ireland (Dublin, *n* = 2; Lisdoonvarna, *n* = 2) and the West of France (Roche, *n* = 1) were placed near to the central Pyrenean samples, the closest of which was an individual from Segre in the Eastern Pyrenees.

**FIGURE 3:**
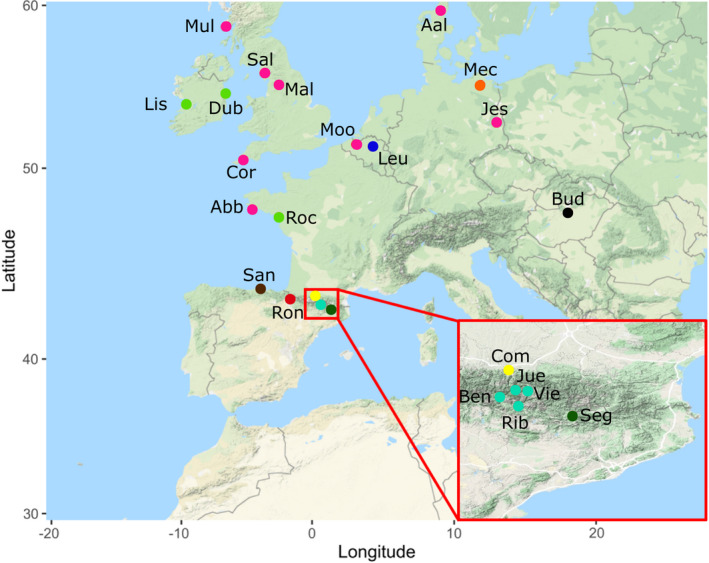
European map showing the locations and abbreviation code for the samples used in this study, with locations coloured according to the groups identified by a cluster analysis of the genomic data

**FIGURE 4:**
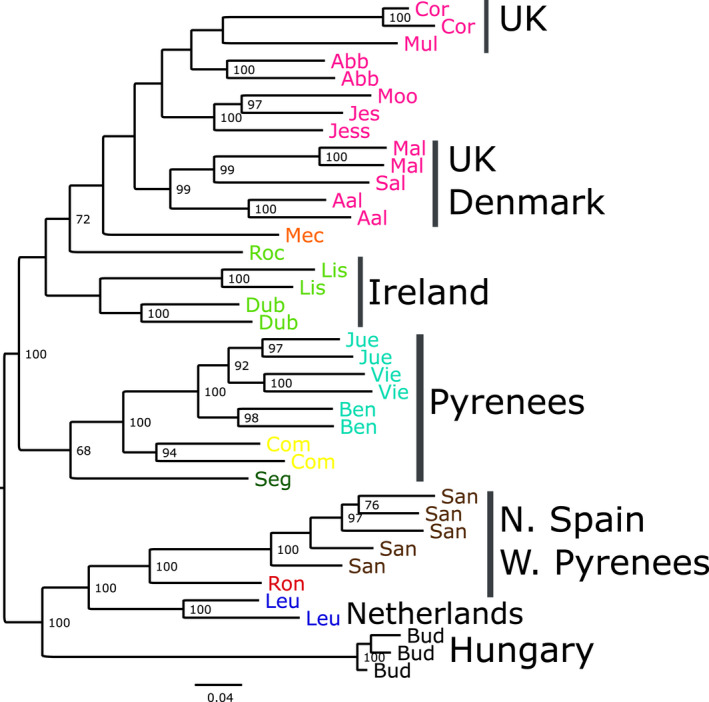
Mid‐point rooted RAxML phylogeny, based on an analysis of 8689 biallelic loci. Bootstrap support >70% is shown. Colours are in accordance with the clusters defined by Gaussian finite mixture modelling

Gaussian finite mixture modelling returned 10 clusters as the best‐fitting model (EII, spherical, equal volume; BIC 349.23 and ICL 349.22; *p* < 0.001 compared with the next best model); Figures 2, 3, 4 are coloured according to these groupings. Figure 2 shows the clusters in relation to the principal component analysis; Figure 3 shows the geographic distribution of three clusters.

A RAxML phylogeny (Figure 4) based on the whole‐genome data mainly corroborated the principal components analysis in recovering a Northern Spanish and West Pyrenean group (Santander and Roncesvalles), a Hungarian group, a central European group (French, German, Danish and British) and multiple lineages in the central and Eastern Pyrenees. There were some populations/individuals that did not fall in this general grouping, including snails from Leuven in Belgium (*n* = 2) and Mecklenburg in Germany (*n* = 1). Although the phylogeny shows some well‐resolved groups, the deeper structure is not well supported.

**FIGURE 5:**
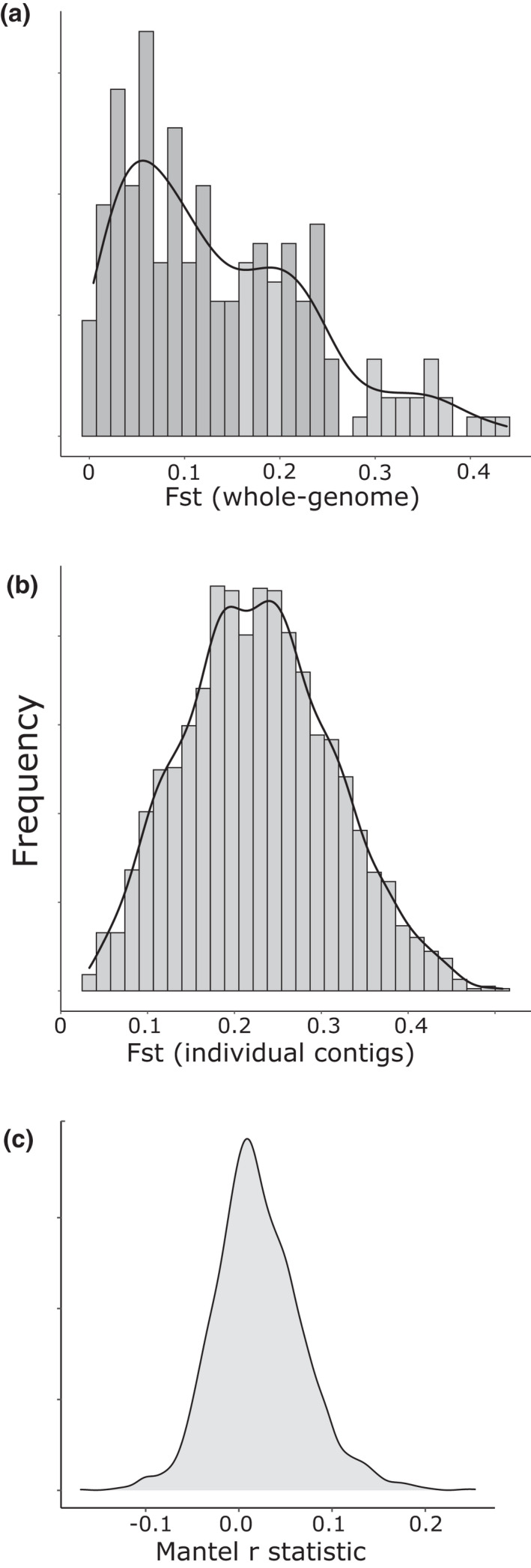
Histograms showing how genetic differentiation varies between individuals and between loci. (a) Estimates of *F*_st_ between individuals based on whole‐genome data. (b) Estimates of *F*_st_ between individuals for each of the 2323 genome contigs (c) estimated mantel *r* statistic, derived by testing the correlation of *F*_st_ for each of the 2323 genome contigs against geographic distance, using Euclidean Haversine distances

**FIGURE 6:**
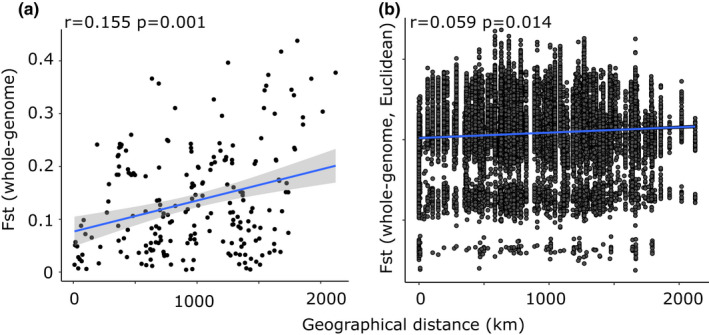
Correlations of *F*_st_ between individuals versus geographic distance using the ddRAD data, a) shows the *F*_st_ (averaged across all loci) versus geographic distance. b) Shows the same data but transformed to represent a Euclidian genetic distance and geographic Haversine distance

Across all pairwise population comparisons, the mean genome‐wide *F*_st_ was 0.14, but with a wide range of individual values (0.00 to 0.44; SD = 0.10; Table 2). The frequency distribution revealed around three groups, with peaks of *F*_st_ at approximately 0.05–0.015, 0.2 and 0.3 (Figure 5a). The highest structure was between samples from Budapest, Hungary and elsewhere (*F*_st_ > ~0.3), with the next‐most divergent samples those from Santander, Spain and elsewhere (*F*_st_ ~ 0.2). There was a shallow, but significant relationship of *F*_st_ by geography (Figure 6), irrespective of the precise method used.

**TABLE 2:** Mean *F*_st_ across all loci; values are coloured according to whether they are between 0.2 and 0.3, or >0.30

		Leu	Moo	Aal	Cor	Mal	Sal	Roc	Abb	Jes	Mec	Bud	Dub	Lis	Mul	Ben	Com	Jue	Ron	San_1	San_2	Seg	Vie
Belgium	Leuven																						
	Moortsele	0.02																					
Denmark	Aalborg	0.11	0.06																				
England	Cornwall	0.20	0.06	0.22																			
	Malham	0.18	0.04	0.13	0.26																		
	St. Bees	0.06	0.08	0.02	0.11	0.01																	
France	La Roche	0.04	0.11	0.01	0.06	0.04	0.09																
	Pont L'Abbe	0.10	0.09	0.03	0.03	0.13	0.03	0.07															
Germany	Jessern	0.06	0.11	0.02	0.15	0.11	0.06	0.05	0.02														
	Mecklenburg	0.01	0.36	0.03	0.09	0.05	0.10	0.11	0.05	0.24													
Hungary	Budapest	0.33	0.30	0.40	0.44	0.42	0.33	0.30	0.35	0.37	0.31												
Ireland	Dublin	0.10	0.03	0.08	0.18	0.13	0.05	0.07	0.04	0.05	0.03	0.37											
	Lisdoonvarna	0.15	0.05	0.16	0.23	0.19	0.06	0.01	0.14	0.12	0.06	0.38	0.02										
Scotland	Mull	0.14	0.11	0.07	0.06	0.04	0.17	0.14	0.09	0.10	0.13	0.30	0.09	0.08									
Spain	Benasque	0.16	0.01	0.15	0.24	0.24	0.06	0.01	0.08	0.09	0.03	0.37	0.10	0.15	0.08								
	Commitges	0.12	0.00	0.12	0.18	0.17	0.03	0.02	0.07	0.08	0.02	0.31	0.04	0.09	0.04	0.02							
	Jueu	0.17	0.07	0.17	0.24	0.21	0.09	0.05	0.14	0.12	0.08	0.35	0.11	0.15	0.05	0.06	0.01						
	Roncesvalles	0.03	0.19	0.12	0.20	0.16	0.22	0.17	0.10	0.26	0.16	0.29	0.22	0.28	0.17	0.11	0.07	0.14					
	Santander_1	0.17	0.20	0.23	0.25	0.23	0.21	0.18	0.18	0.21	0.17	0.29	0.20	0.21	0.21	0.22	0.18	0.24	0.05				
	Santander_2	0.17	0.20	0.24	0.25	0.24	0.21	0.19	0.19	0.21	0.17	0.28	0.19	0.22	0.20	0.22	0.20	0.24	0.05	0.01			
	Segre	0.06	0.02	0.07	0.13	0.10	0.01	0.04	0.03	0.03	0.01	0.25	0.03	0.00	0.04	0.10	0.07	0.03	0.03	0.19	0.19		
	Vielha	0.16	0.06	0.15	0.23	0.21	0.08	0.04	0.12	0.11	0.06	0.34	0.09	0.14	0.04	0.05	0.03	0.05	0.11	0.24	0.24	0.09	

In individual contigs, there was considerable variation of *F*_st_, showing a mean of 0.23 across the whole genome (range 0.03 to 0.51; SD = 0.09; Figure 5b). To further understand geographic and genomic variation, we estimated Mantel's *r* statistic for the *F*_st_ for each contig and all pairwise comparisons of populations. Although the mean *r* was greater than zero (Figure 5c; mean = 0.021), only ~1/3 of the genome contigs showed a significant association with geography.

**FIGURE 7:**
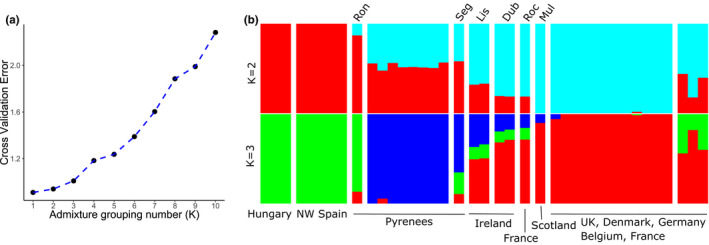
Population clustering using ADMIXTURE. a) Output of cross‐validation assay. b) Population clustering using K = 2 and K = 3. Each vertical bar represents an individual, with the proportion of each colour representing the inferred ancestry

Admixture analyses were used to test the hypothesis that some populations are descended from introgression between Pyrenean and other European snails (Figure 7). For both *K* = 2 and *K* = 3, individuals from Ireland, especially the West coast (Lisdoonvarna) showed mixed ancestry between the Pyrenean snails and elsewhere. Samples from the East coast of Ireland (Dublin), and possibly also the North West coast of France (La Roche) and the Scottish island of Mull, may also show some evidence of mixed ancestry, albeit to a less degree (Figure 7).

D‐statistical analyses were used to further explore the patterns of admixture (Table S5). Specifically, we set out to test whether the Irish samples (Lisdoonvarna, Dublin) and some others (e.g. La Roche, Mull) show evidence of introgression from other populations. In general, there was no evidence for an excess of allele sharing (Table S5, Z‐score between −3 and 3) and therefore, no evidence for introgression since their common ancestry. The main exception was a significant enrichment of shared sites between Segre in the Eastern Pyrenees and Lisdoonvarna in Ireland, irrespective of whichever central European site used (Z‐score around −5 to −6). This is consistent with these West Irish samples being partly derived from snails in the Eastern Pyrenees (Table S5). There was also limited evidence that the Dublin population may be partly derived from Pyrenean snails and the same for samples from La Roche (*Z*‐score < −3 in two comparisons each). These results are, therefore, consistent with Irish and possibly some French populations being derived from an introgression event between Pyrenean and central European populations of snails. In the future, it should be possible to better test for evidence of introgression, using analyses such as treeMix (Pickrell & Pritchard, 2012), but unfortunately, a relatively high degree of missing data precluded this analysis here.

## 4 DISCUSSION

Investigating the phylogeography of *Cepaea nemoralis*–and the relative degree of gene flow between populations—should be an important step towards understanding the relative roles that natural selection and drift have to play in the evolutionary history of the colour and banding polymorphism. In this study, we used a newly available draft genome (Saenko et al., 2021) and the ddRAD method, supplemented with a wide geographic survey of mtDNA data, with a primary aim to begin to understand the post‐glacial history of the species, but also to provide context for future studies on the evolutionary origins of the supergene, and the roles that selection and drift have in determining the local colour polymorphism. Of course, these methods are now standard in many animal groups, but the study is relatively novel for snails, partly because molluscs in general lag behind (Davison & Neiman, 2021), with, for example, only a few other land snail species having a genome assembly (Chueca et al., 2021b; Guo et al., 2019; Liu et al., 2021). The reality is that although phylogeographic and genomic studies are relatively common in other invertebrates, there are remarkably few similar studies in snails or even the wider group of molluscs.

Broadly, analysis of the whole‐genome data expands and adds nuance to the mtDNA survey. The analysis shows that European‐wide patterns of differentiation are primarily due to multiple deeply divergent groups of snail. Geographically one group is dominant, being found over much of Central Europe (Figures 1 and 2). There are also likely multiple Italian populations, some of which may have colonized to the limits of the eastward distribution of the species (e.g. in Croatia and newly colonized Budapest). Finally, there are multiple lineages in Iberia, roughly centred along the Northern coast and Cantabrian mountains, with separate populations in regions of the Pyrenees. In the future, further sampling, especially in potential refugia in the Balkans and Italy, may reveal other divergent groups, some of which may have very localized distributions.

The results corroborate previous studies in that populations showed a high degree of genetic structuring, sometimes over short geographic distances (Davison & Clarke, 2000). For example, the maximal genomic distance was between snails in Hungary and the rest of Europe (*F*_st_ > 0.3; Table 2; Figure 5). In comparison, samples from the UK, Denmark, Germany, France and Belgium were relatively homogenous, with an *F*_st_ around 0.05 to 0.15. Previously, Ochman et al. (1983) showed using allozymes that the Pyrenees contain three deeply differentiated sets of populations, or ‘molecular area effects’, for which the geographic patterns did not correlate with the shell ground colour and banding frequencies. The genomic evidence also supports these findings, showing deep divergence between geographically close populations on the Atlantic coast of Northern Spain (e.g. Santander), and the Central Pyrenees (e.g. Vielha) with possible hybrid populations in both the Western and Eastern Pyrenees (e.g. Roncesvalles in West and Segre in East).

### 4.1 Origins of the Irish *Cepaea nemoralis*

One of the key characteristics of most population genetic studies of land snails is that they show high population structure, and deeply diverged mitochondrial lineages, the latter frequently considerably <5% *within* species (Chiba, 1999; Thomaz et al., 1996), for which a variety of explanations have been put forward (Davison, 2006; Parmakelis et al., 2013). In *Cepaea*, one of the most striking findings from previous work was that some Irish *C. nemoralis* are most likely derived from a post‐glacial colonization event from the Pyrenees (Grindon & Davison, 2013), in part because of identical mitochondrial haplotypes in the two locations.

Here, the genomic data provide further evidence for the long‐distance colonization, but also show that the present‐day snails in Ireland are likely descendants of admixture between Pyrenean snails and individuals from the population that colonized most of Central Europe. The snails from the West coast of Ireland show the greatest proportion of Pyrenean ancestry (Figure 7; Table S5), consistent with the observation that they also have some of the same shell characters, including large size and shells with a pale or white lip colour (Cameron, 1969; Cook & Peake, 1960, 1962). The analyses may also indicate, albeit a lesser signal, of this same admixture in snails from Dublin, Ireland, and the North‐West of France (La Roche; Figure 7; Table S5). As there is also another mitochondrial DNA lineage present in Ireland (D), which may derive from Northern Spanish or West Pyrenean snails (directly or indirectly), then Ireland may have been colonized on at least two and perhaps three or more occasions.

Similar patterns of mixed ancestry have been reported for other species (especially those involving the ‘Celtic fringe’; Brace et al., 2016; Kotlik et al., 2018; McDevitt et al., 2011; Searle et al., 2009). Altogether, these new results contribute to the ongoing debate regarding the uncertain origins of the Irish biota (e.g. McDevitt et al., 2020).

Although not proven, we believe that the most likely scenario is that *C. nemoralis* snails arrived directly in Ireland from the Pyrenean region, intentionally or accidentally assisted by humans. For the moment, the precise geographic origin remains unknown—identical mtDNA haplotypes are shared between multiple sites in the Pyrenees and Ireland, with mtDNA *F*_st_ values not significantly different from zero (Grindon & Davison, 2013). In the genomic study here, a single site (Segre) in the Eastern Pyrenees falls most closely with the Irish snails. However, this population in itself could be a hybrid population, with the actual Irish ancestor elsewhere.

It is reasonable to suppose that snails of the same lineage could also have been distributed along the western fringes of France. Of use here, an inspection of the data in Layton et al. (2019) shows that snails of mtDNA lineage C are found in central and North Western France (see their Figure 1; Deux‐Sevres, Paimpont), whereas previously lineage C was reported from the Pyrenean region and Ireland only (Grindon & Davison, 2013). This finding is, therefore, consistent with the genomic evidence (especially the admixture analyses) that indicates a possible hybrid origin of snails in North‐West France (La Roche; Table S5). However, the specific mitochondrial C type haplotype found in France has not yet been found in Ireland. It is, therefore, feasible that these populations in Ireland and France are the result of separate admixture events.

In terms of dating, fossil material indicates that *Cepaea nemoralis* may have colonized Britain first, perhaps 1000 years before Ireland, the latter of which was at least 8000 years ago (Newlands Cross, Co. Dublin: 7600+/−500 BP Cartronmacmanus, Co. Mayo: 8207+/−165; Kerney et al., 1980; Preece et al., 1986; Speller, 2006). Assuming that these relative dates are correct, then a new arrival of *C. nemoralis* in Ireland would have quickly become established in the vacant habitat. In comparison, in Britain, new arrivals would have had to compete with more numerous existing snails and so perhaps only became established on the fringes of the distribution. Populations have since hybridized and introgressed. In perhaps a similar manner, founding populations of mice were likely transported by humans (Jones et al., 2013). Moreover, *C. nemoralis* are arguably ‘synanthropic’, liking disturbed habitats but also being consumed as food source, especially in Iberia, since at least 11 000 years before the present (Gutiérrez Zugasti, 2011), and they are commonly found as an item of food refuse in Mediterranean archaeological sites just prior to the onset of agricultural economies (although not in Ireland – Eva Laurie, pers. comm.). Recently founded populations of *C. nemoralis* also illustrate the ease with which they may be introduced by humans, especially in disturbed habitats, including in much of Poland (Ożgo et al., 2019), and parts of Sweden (Cameron & von Proschwitz, 2020), the Czech Republic (Peltanová et al., 2012), Hungary (Budapest; Szili‐Kovács et al., 2012) and Russia (Moscow; Egorov & Sverlova, 2021).

### 4.2 Colour and genomic geographic variation

One of the characteristics of the genus *Cepaea* is that all, or nearly all, populations show some degree of colour or banding polymorphism (Cook, 2017; Jones et al., 1977). Nonetheless, large‐scale and local surveys of the geographical distribution of shell ground colour have shown clear geographical patterns (Cameron & Cook, 2012; Cook, 2014; Davison et al., 2019; Ożgo & Schilthuizen, 2012; Ramos Gonzalez & Davison, 2021; Silvertown et al., 2011; Worthington et al., 2012). Future studies that aim to test trends in shell variation may, therefore, wish to take account of this knowledge in analyses. For the moment, the whole‐genome data enable a more nuanced understanding, for example, showing that large scale differentiation due to isolation by distance is mainly driven by about a third of the genome (Figure 3c).

To what extent is the lack of geographic differentiation for most loci due to neutral processes such as drift (enabled by gene flow), or else because of a selective sweep? Unfortunately, the relatively low contiguity of the genome assembly and the relatively shallow ddRAD sampling means that the present data set lacks power for more sophisticated analysis, such as a genome scan. It is, therefore, premature to understand the relative contribution of chance events, gene flow or selection in explaining patterns at individual loci. In the future, we hope that it should be possible to identify the genes involved in determining the polymorphism, in which case it would be fruitful to compare associations of colour alleles with geography. For the moment, it is our impression that the yellows, pinks and even browns of many Pyrenean (and Irish) populations are somewhat different—more ‘exuberant’ as Franks and Oxford (2009) might put it—compared with other populations (Figure 1b). Although only speculation, a possible explanation is that the alleles for colour in the separate populations have also diverged, which should be evident in any phylogenies based on colour‐linked loci.

In some other species, such as *Heliconius* butterflies, phylogenies based on colour‐linked loci show strong associations with the wing colour. This is especially the case when markers are tightly linked and occur irrespective of the species (e.g. Pardo‐Diaz et al., 2012). As our previous work showed that colour is ‘indiscrete’ or continuous, and not three separate colours in *Cepaea* (Davison et al., 2019), further sequence analysis of colour alleles will inform how selection has impacted upon the genome and more specifically, the nature of this selection.

### 4.3 The evolutionary history and future of *Cepaea*

A recent revision of the taxonomy means the genus *Cepaea* now contains only two species, *C. nemoralis* and *C. hortensis*, with *C. sylvatica* and *C. vindobonesis* removed to another genus, *Caucasotachea* (Kajtoch et al., 2017). As the known sister taxa to *Cepaea* are Iberian (Neiber et al., 2016, 2021; Neiber & Hausdorf, 2015a), then a guess is that the ancestor of *Cepaea* itself may have originated in Iberia, subsequently colonizing the rest of Europe. Unfortunately, the genomic data presented here do not easily inform on the origination of the genus or the evolution of the polymorphism. The mitochondrial DNA data on their own are derived from the short ‘barcoding’ region, with potential outgroups to *Cepaea* rather distant. In consequence, although the phylogeny (Figure S1) indicates that lineages from Italy may be basal, this positioning is dependent upon an outgroup which may not be appropriate.

In comparison, the data presented here are more informative of relatively recent events. Certainly, there are multiple divergent lineages in Iberia (mtDNA and genome), which could equally imply the origination of the species there, but caution is required because there are also divergent populations and lineages in other likely refugia, especially Italy/Croatia (mtDNA) and Hungary (ddRAD—population recently established and so must be derived from elsewhere?). Also, although this and the previous work (Grindon & Davison, 2013) show that much of mainland Europe was likely colonized by snails with mtDNA lineages A and B, the actual refugial location(s) are not clear. Further fine sampling is required in Italy, the Balkans, Alps, Mediterranean coast, western coast of France and the valleys of the Pyrenees. For the moment, on the basis of the evidence provided here, and the distribution of samples, it seems plausible that the central European population is derived from an as yet unsampled refugium in the Italian or alpine region, or perhaps adjacent Balkans—which would make the pattern most similar to that of the *Chorthippus parallelus* paradigm suggested by Hewitt (2004).

In the future, we believe that a higher level assembly of the *C. nemoralis* genome should enable subsequent studies on the evolutionary origins of the supergene, and the relative roles that natural selection, recombination and drift may play in the establishment and loss of colour polymorphism in species as a whole and, more specifically, local populations. The same phylogeographic and population genomic questions should also be addressed in the sister species *C. hortensis*, which has a more northerly distribution; many records for *C. hortensis* in the Iberian Peninsula are arguably erroneous, instead of being pale‐lipped *C. nemoralis* (Puente et al., 1998, Ramos Gonzalez & Davison, 2021; see also records in e.g. iNaturalist). This species also colonized North America, likely more than 6000 years ago (Pearce et al., 2010), and is long present in Iceland, and perhaps once present in Greenland (Johnson, 1906). It is not known how this species colonized these locations, but population genomics would reveal whether it was from one or several sources and whether a ‘stepping‐stone’ was involved (e.g. Europe – Iceland – North America).

## CONFLICT OF INTEREST

The authors declare no competing interests.

## Supporting information


Figure S1

Figure S2

Tables S1–S5

## Data Availability

The Illumina ddRAD reads for each individual are available via the NCBI Bioproject accession PRJNA730503. New mtDNA haplotypes have Genbank accession numbers ON556571‐ON556582. The mitochondrial alignment, ddRAD vcf files and phylip file used for the analyses are available in the Dryad database https://doi.org/10.5061/dryad.brv15dvc5. Some simple scripts are available on https://github.com/angusdavison.
