# Supplementary figures and images for: Deep structure, long‐distance migration and admixture in the colour polymorphic land snail Cepaea nemoralis
Source: J Evol Biol. 2022 Jul 13;35(8):1110–25. doi: 10.1111/jeb.14060 (PMC9541890; doi:10.1111/jeb.14060)

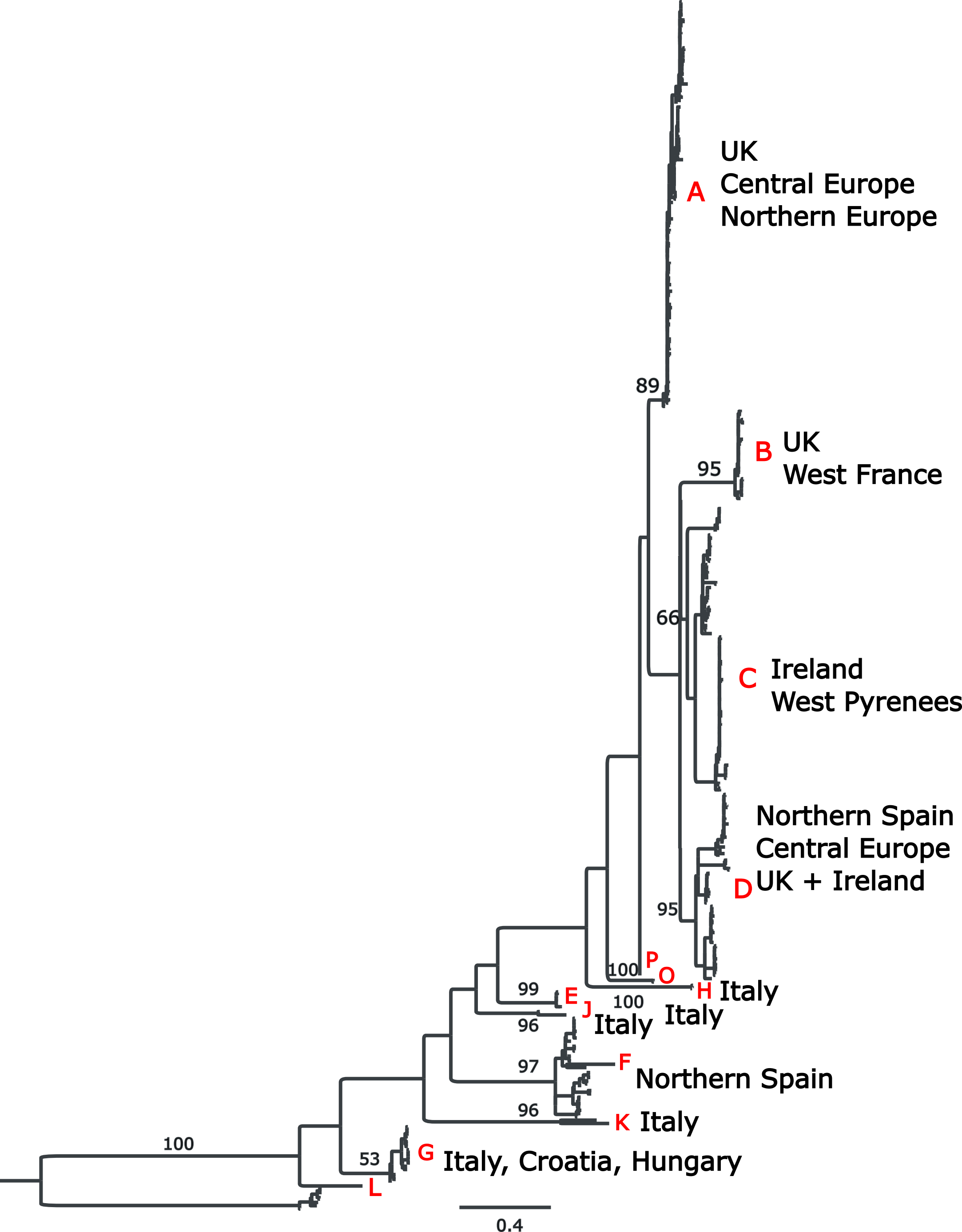

Supplement: Supplementary file 1 — Figure S1 [file JEB-35-1110-s001.pdf]

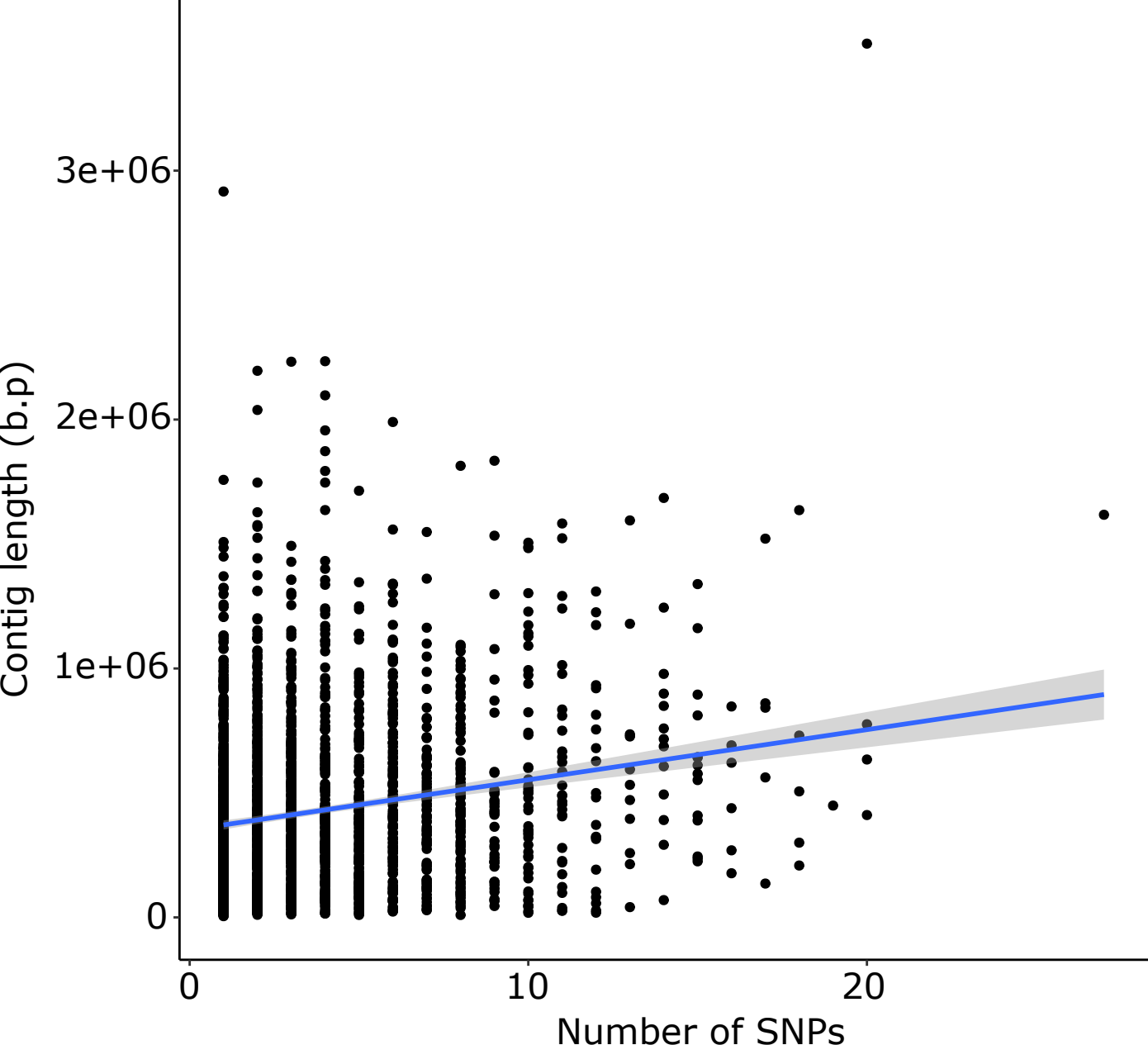

Supplement: Supplementary file 2 — Figure S2 [file JEB-35-1110-s003.pdf]
